# Trace Analysis in End-Exhaled Air Using Direct Solvent Extraction in Gas Sampling Tubes: Tetrachloroethene in Workers as an Example

**DOI:** 10.1155/2014/904512

**Published:** 2014-03-18

**Authors:** Chris-Elmo Ziener, Pia-Paulin Braunsdorf

**Affiliations:** Federal Institute for Occupational Safety and Health (BAuA), Group 4.2 “Biomarkers”, Nöldnerstrasse 40-42, 10317 Berlin, Germany

## Abstract

Simple and cost-effective analytical methods are required to overcome the barriers preventing the use of exhaled air in routine occupational biological monitoring. Against this background, a new method is proposed that simplifies the automation and calibration of the analytical measurements. End-exhaled air is sampled using valveless gas sampling tubes made of glass. Gaseous analytes are transferred to a liquid phase using a microscale solvent extraction performed directly inside the gas sampling tubes. The liquid extracts are analysed using a gas chromatograph equipped, as usual, with a liquid autosampler, and liquid standards are used for calibration. For demonstration purposes, the method's concept was applied to the determination of tetrachloroethene in end-exhaled air, which is a biomarker for occupational tetrachloroethene exposure. The method's performance was investigated in the concentration range 2 to 20 **μ**g tetrachloroethene/L, which corresponds to today's exposure levels. The calibration curve was linear, and the intra-assay repeatability and recovery rate were sufficient. Analysis of real samples from dry-cleaning workers occupationally exposed to tetrachloroethene and from nonexposed subjects demonstrated the method's utility. In the case of tetrachloroethene, the method can be deployed quickly, requires no previous experiences in gas analysis, provides sufficient analytical reliability, and addresses typical end-exhaled air concentrations from exposed workers.

## 1. Introduction

If a worker is exposed to a workplace chemical, the chemical can enter the body. Analytical determination of the chemical or its metabolites in the body allows estimation of the absorbed dose and ultimately the health hazard. Biological samples such as blood or urine are routinely used for this kind of analysis, which is known as biological monitoring, or biomonitoring for short.

Absorbed volatile chemicals are partly eliminated from the body via exhalation. Exhaled air, also referred to as exhaled breath, is therefore also suitable for analysis in biological monitoring. Nevertheless, exhaled air is rarely used outside of research applications within this specialist field. Possible reasons for this include a lack of practical, reliable, and commercially available sampling systems, as well as analytical difficulties [1].

Against this background, a new exhaled air analysis method is to be developed that overcomes the previous operating limits and can be used in routine situations. Tetrachloroethene was chosen as a model substance for the development process.

Tetrachloroethene (CAS number: 127-18-4; synonyms: tetrachloroethylene, perchloroethylene, PER, PCE) is a volatile solvent widely used in various technical processes and as an intermediate in the chemical industry [2]. Solvent applications are well known in cleaning procedures such us dry cleaning, metal degreasing, and film restoration [2]. Workers are exposed via inhalation and dermal absorption [3]. The majority of the incorporated neurotoxic substance is eliminated unchanged via exhalation [3]. The American Conference of Governmental Industrial Hygienists (ACGIH) and the Scientific Committee on Occupational Exposure Limits (SCOEL) have suggested assessment values for tetrachloroethene in exhaled air from exposed workers [3, 4].

Several sampling and analytical methods have been reported for the determination of tetrachloroethene in exhaled air: for example, exhaled air sampling has been achieved using glass tubes that can be sealed with septum caps [5–8] or valves [9, 10], bags made of polyvinyl fluoride [11–18] or polyvinylidene chloride [8, 19], adsorbent tubes [20, 21], more complex sampling devices [22, 23], or by direct exhalation into an analyser [24, 25]. Sample analyses were performed using a gas chromatograph [5–13, 15–22], surface acoustic wave sensors [14], an atmospheric-pressure ionization mass spectrometer [24], or an infrared spectrometer [8, 25]. The gaseous samples were transferred directly into the measurement device using gas-tight syringes [5–9, 11, 15–19, 22], or the tetrachloroethene was extracted from the breath or breath samples using solid-phase microextraction (SPME) [10] or adsorption tubes and then analysed following thermal desorption [10, 13, 14] or liquid elution [12, 20, 21].

Application of the suggested assessment values for tetrachloroethene requires strict adherence to the specified sampling times “prior to shift” [4] or “prior to the last shift of a work week” [3]. Routine use of exhaled air in biological monitoring therefore requires sampling methods that could be performed by the clients themselves under field conditions if necessary. The requirements for routine use are not currently met by direct-reading instruments, for example, an atmospheric-pressure ionization mass spectrometer [24] or infrared spectrometer [25] coupled with a breath inlet system, or by technically complex sampling systems, for instance, with built-in pumps and a multitude of valves [23].

Exhaled air consists of ambient air retained in the respiratory dead space and alveolar air [26]. The latter has been involved in gas exchange in the lung and can be sampled after the dead space air has been exhaled [26]. For this reason, alveolar air is also called end-exhaled air. The quoted assessment values for tetrachloroethene are defined explicitly for end-exhaled air. The breath-sampling method must therefore ensure that only this exhaled air fraction is sampled.

For routine uses, the most appropriate sampler seems to be a valveless glass tube that can be sealed with septum caps. Due to their design, such tubes sample end-exhaled air if the tube volume is less than the alveolar air volume. For sampling, a subject needs only to exhale once completely through the open tube. Tubes of this kind allow self-sampling [27], are inexpensive to manufacture, and do not allow gas to permeate through their glass wall. Glass tubes equipped with valves can be sealed very quickly after sampling, but valves make the sampling device bulky and expensive for the purposes of routine sampling.

In contrast to glass tubes, bags do not collect end-exhaled air automatically [26]. The subject therefore has to exhale the dead-space air into the environment and then the alveolar air into the bag [18]. The sampling procedure therefore seems error-prone, and the results are more strongly influenced by the subject's cooperation.

Direct exhalation into adsorption tubes is well suited to analyte enrichment and stabilization of gaseous samples for transport and storage. However, the sampling procedure requires that the sampled volume be measured using an additional device, which reduces the routine practicality of the method.

To overcome the current barriers to routine exhaled air analysis, a proposed method must take into account the technical realities of typical biomonitoring laboratories. The basic equipment of such laboratories often includes a gas chromatograph coupled with a liquid autosampler. In contrast, the handling of adsorbent tubes and thermal desorption techniques is more common in air-monitoring than biomonitoring laboratories. The latter are familiar with solid-phase microextraction techniques, but the use of such techniques for routine breath analysis requires sophisticated automation solutions. The same applies to direct injection of breath samples into the analyser. Furthermore, calibration is carried out using gaseous standards in both cases. Unfortunately, biomonitoring laboratories are not usually familiar with gas calibrations.

Liquid sample analyses are one strength of biomonitoring laboratories, which commonly analyse blood and urine samples. Solvent extraction of analytes from exhaled air samples allows direct transformation of the gaseous samples into “liquid samples” and, consequently, the use of existing skills and technologies. The process calibration can therefore be performed with liquid standards, and a typical liquid autosampler can be used for the automation of the measurement step. In conclusion, the method presented in this work was conceived based on sampling end-exhaled air using valveless glass tubes, solvent extraction of the analyte, and automated liquid-sample analysis using gas chromatography. To the authors' knowledge, no end-exhaled air analysis methods have yet been published that use direct solvent extraction.

## 2. Experimental

### 2.1. Exhaled Air Analysis

#### 2.1.1. Sampling

**Figure 1:**
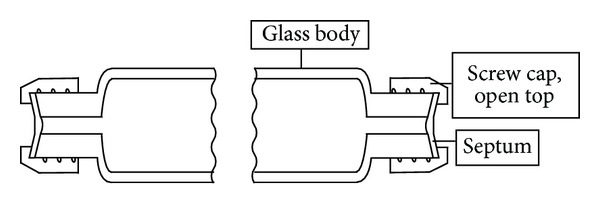
Sketch of the valveless gas sampling tube, sealed with septum caps.

*(1) Exhaled Air Sampler*. Breath samplers of the valveless glass-tube type were not commercially available. An internally developed sampler was therefore used (Figure 1). The valveless exhaled air sampler, similar to the description by Stewart [28], consisted of a glass tube (outer diameter 2 cm, length 20.5 cm) with threads (thread size 13–425) on both ends and two open-top septum screw caps. Unlike in Stewart's work, however, not only were the tube dimensions changed, but the glass tube openings were also optimized. The tube openings on both ends were formed as cylindrical holes (inner diameter 3 mm, length 10 mm). The glass tubes were custom-manufactured to our specific requirements by Glastechnik Gräfenroda GmbH (Gas Sampling Tube—Type BAuA, Gräfenroda, Germany).

**Figure 2:**
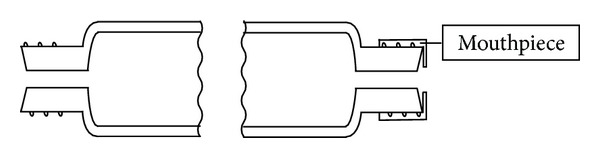
Sketch of the valveless gas sampling tube, ready for sampling; open-top cap screwed on as a mouthpiece.

The screw caps were made of glass-filled nylon in a robust design (thread size 13–425, Kimble Chase, Rockwood, USA) and contained PTFE-lined silicone septa (75 mils thick, Supelco, Bellefonte, USA). The volume of a sealed tube was approximately 37.5 mL and was determined gravimetrically for each tube. Open-top screw caps for autosampler vials (thread size 13–425, wide mouth, Infochroma, Zug, Switzerland) without a septum were used as disposable mouthpieces (Figure 2).

**Figure 3:**
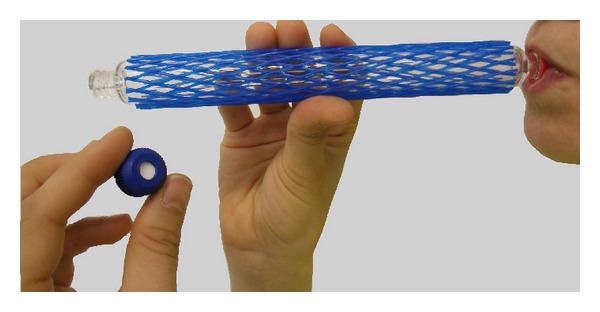
Sampling end-exhaled air with a gas sampling tube; mouthpiece screwed on; left hand: one of two sealing caps with septum; plastic net sleeve to protect glass.

*(2) End-Exhaled Air Sampling Procedure*. For sampling, the subjects breathed normally and then exhaled completely through the glass tubes (Figure 3) after inhaling and holding their breath for 5 seconds. The subjects then removed the mouthpieces and screwed the sealing caps onto the tubes. Samples collected in the field were transported to the laboratory at ambient temperature and in the dark. The glass tubes were protected using plastic net sleeves to ensure safe handling and transport (Figure 3).

#### 2.1.2. Sample Analysis

**Figure 4:**
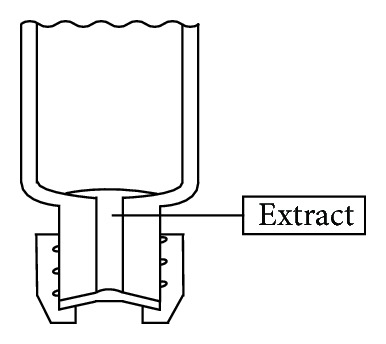
Sketch of the lower side of a gas sampling tube in the vertical position: the exhaled breath extract (the isooctane phase) is located within the cylindrical hole.

*(1) Sample Preparation*. The analyte tetrachloroethene was extracted from the exhaled air samples using a microscale solvent extraction procedure: 200 *μ*L isooctane (Suprasolv, Merck, Darmstadt, Germany) was injected into the breath samples in the sealed gas sampling tubes using a 250 *μ*L syringe (Series G, ILS, Stützerbach, Germany). The tubes were then placed horizontally on an RM10-V 30 tube roller mixer (Labortechnik Fröbel, Lindau, Germany) for 20 minutes at a speed of 1 revolution per minute. After mixing, the tubes were placed in a vertical position with the intact septa at the bottom for 15 minutes to allow phase separation.  Figure 4 shows the separated isooctane phase of the exhaled air extract; it is located within the cylindrical hole of the gas sampling tube.

**Figure 5:**
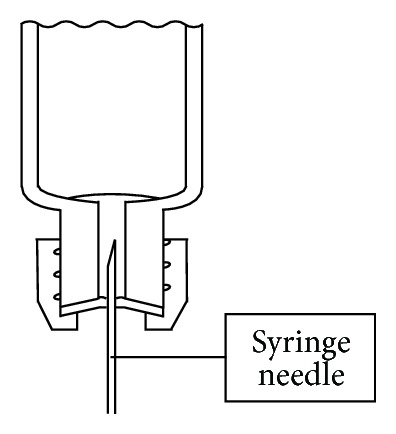
Withdrawing the extract.

The extract was withdrawn from the sealed tubes using a 250 *μ*L syringe (Series G, ILS, Stützerbach, Germany) as shown in Figure 5 and then injected directly into 250 *μ*L glass microvials (iV2 *μ*-Vial, Glastechnik Gräfenroda, Gräfenroda, Germany). The vials were sealed immediately with PTFE-silicone septum screw caps (MS Pure, Glastechnik Gräfenroda, Gräfenroda, Germany) and placed in the autosampler tray of the gas chromatograph for analysis.

*(2) GC/MS Analysis*

*GC-MS System.* GC-MS analysis was performed on a 6890 N gas chromatograph coupled to a 5973 N mass selective detector (Agilent Technologies, Palo Alto, USA), equipped with an MPS2 autosampler (Gerstel, Mühlheim, Germany), and controlled with a ChemStation (Agilent Technologies) and the embedded Maestro software (Gerstel). The gas chromatograph was fitted with a split/splitless injector (split liner: cup design, unpacked, Supelco, Bellefonte, USA) and an HP-1 ms capillary column (30 m × 0.25 mm × 0.25 *μ*m, coated with cross-linked and bonded 100% dimethyl polysiloxane; Agilent Technologies, Palo Alto, USA). Sampling and injection were performed using the autosampler in the syringe-based liquid sampling mode with a 10 *μ*L syringe (Gerstel, Mühlheim, Germany).

*GC-MS Method*. The autosampler injected 2 *μ*L of a sample with a split ratio of 1 : 20. After the injection, the syringe was washed using n-hexane (puriss., absolute, Sigma Aldrich, Munich, Germany) to avoid carryover effects. The gas chromatograph temperature settings were as follows: injection temperature was 250°C; column oven temperature programme was 40°C for 2 min, followed by an increase to 60°C at 5°C/min and then to 90°C at 30°C/min; the transfer line was set to 250°C. Helium 6.0 was used as a carrier gas at a constant flow rate of 1 mL/min. The mass selective detector was operated with electron impact ionization (70 eV) in selected ion monitoring (SIM) mode. Tetrachloroethene was monitored using the target ion 166* m/z* and the qualifier ion 131* m/z*. The target ion was used for quantitation. The analyte was identified using the retention time and the abundance ratio of qualifier ion to target ion. Peak integration was performed using the ChemStation software. The position of the isooctane peak was explored using the ion 99* m/z*; the solvent delay was then set to 4 minutes.

*(3) Calibration*. For calibration, 200 *μ*L of calibration solution (isooctane spiked with tetrachloroethene) was injected into gas sampling tubes containing end-exhaled air from subjects not exposed to tetrachloroethene. The resulting calibration samples were treated the same as real samples after the addition of isooctane according to Section 2.1.2(1) and analysed as described in Section 2.1.2(2). The exhaled air from the subjects who gave the matrix samples was checked for blank values. Calibration curves were obtained by plotting the peak areas of tetrachloroethene as a function of the concentrations or masses used. The latter allowed the actual sample volumes to be taken into account. The determined tetrachloroethene masses were therefore divided by the sample volumes, which corresponded to the volumes of the sampling tubes used.

*Preparation of Calibration Solutions*

*Stock Solution I*. 30 *μ*L of tetrachloroethene (analytical standard, Sigma-Aldrich, Steinheim, Germany) was drawn into a 50 *μ*L syringe. The standard was injected into a 10 mL volumetric flask partly filled with isooctane (Suprasolv, Merck, Darmstadt, Germany). The syringe was weighed before and after injection to determine the mass of standard injected. The flask was made up to the mark with isooctane.

*Stock Solution II*. A 200 *μ*L aliquot of stock solution I was pipetted (200 *μ*L, variable, Eppendorf Research plus, Eppendorf, Hamburg, Germany) into a 10 mL volumetric flask and diluted to the mark with isooctane.

*Calibration Solutions*. Microlitre volumes of stock solution II were aliquoted into 10 mL volumetric flasks using adjustable pipettes (Eppendorf Research, Eppendorf) and diluted to the mark with isooctane. The aliquots of stock solution II were calculated such that 200 *μ*L of each calibration solution corresponded to the required tetrachloroethene concentration in the gas sampling tube.

All solutions were stored at 4°C in 10 mL capillary bottles (Certan, Sigma Aldrich, Steinheim, Germany), where they remained stable for at least one week.

### 2.2. Method Performance Evaluation

#### 2.2.1. Calibration

For the purpose of method evaluation, a 10-point calibration was established in the concentration range 2 to 20 *μ*g tetrachloroethene/L exhaled air. The calibration samples were prepared and analysed as described in Section 2.1.2(3).  Table 1 shows an example pipetting scheme.

#### 2.2.2. Method Precision

To determine the precision of the method, spiked end-exhaled air samples (*n* = 10 in each case) were analysed intraday at the concentrations levels 4 and 15 *μ*g tetrachloroethene per litre according to Section 2.1.2. The spiked samples were prepared as follows: end-exhaled air was obtained according to Section 2.1.1 from a subject not exposed to tetrachloroethene and spiked with tetrachloroethene standard gas (10 *μ*L or 37 *μ*L) using gas-tight syringes (1701N/1705N series, Hamilton, Bonaduz, Switzerland). To keep the septa of the gas sampling tubes intact, the screw caps were unscrewed at one end for gas injections and then screwed back on quickly.

The standard gas was prepared using a static method: using a 25 *μ*L syringe, 20 *μ*L of tetrachloroethene (analytical standard, Sigma Aldrich, Steinheim, Germany) was injected into a 2.2 L static dilution bottle with a valve (Sigma Aldrich, Bellefonte, USA). The syringe was weighed before and after injection to determine the mass of standard injected. The exact bottle volume was determined gravimetrically. The bottle was stored overnight at room temperature to evaporate the substance fully and to equilibrate the gas concentration.

#### 2.2.3. Accuracy

Spiked end-exhaled air samples, prepared as described in Section 2.2.2, were used for recovery experiments. These samples—10 at each of the concentration levels 4 and 15 *μ*g/L tetrachloroethene—were analysed according to Section 2.1.2.

#### 2.2.4. Storage Stability of Breath Samples

The storage stability of end-exhaled air samples was determined using spiked samples at the concentration levels 4 and 15 *μ*g/L. Twenty samples at each concentration were prepared as described in Section 2.2.2. In each case, 10 samples were analysed according to Section 2.1.2 on the day of their creation and 10 samples were analysed after one week of storage in the dark at room temperature.

#### 2.2.5. Limits of Detection and Quantification

The limits of detection and quantification were determined using the calibration curve procedure, following the description by Bader et al. [29]. Calibration standards were prepared in end-exhaled air according to Section 2.2.2. However, the standard gas was diluted beforehand as follows: 5 mL of the standard gas was injected in a second static dilution bottle and the resulting gas was used as the spiking gas. Ten equidistant calibration points in the concentration range 0.005 to 0.05 *μ*g tetrachloroethene/L were analysed according to Sections 2.1.2(1) and 2.1.2(2); the lowest concentration point was close to the expected detection limit.

#### 2.2.6. Field Study

End-exhaled air analyses were performed on four dry cleaners (two men, two women) with known exposure to tetrachloroethene while working in a dry-cleaning shop and a control group of 10 subjects (five men, five women) without such exposure. The dry-cleaning shop used tetrachloroethene as the cleaning solvent and worked primarily with leather garments. The employees' work tasks were as follows: operating machines, pressing, dyeing, and tagging/inspection. The ethics committee of the Berlin Chamber of Physicians approved the study protocol. All subjects signed informed consent forms.

End-exhaled air sampling was carried out as described in Section 2.1.1 and, in the case of the exposed workers, on a Friday prior to the last shift of the working week. All subjects filled two gas sampling tubes for tetrachloroethene analysis using the proposed method. Samples were collected outdoors to avoid contamination. The ambient temperature and atmospheric pressure were recorded at the sampling site at the moment of sampling. The samples were transported to the laboratory, stored in the dark and at room temperature over the weekend, and analysed on Monday as described in Section 2.1.2. A calibration was performed on the day of measurement for the purposes of quantification. In addition, the mass of water in each of the tubes was determined gravimetrically by weighing the tubes before and after sampling.

## 3. Results and Discussion

### 3.1. Method Development—Basics

#### 3.1.1. End-Exhaled Air Sampler

The end-exhaled air sampler used in sampling was self-developed in the form of a valveless gas sampling tube made of glass and sealable with septum caps (Figure 1). The tube openings were designed as cylindrical holes with a diameter of 3 mm. The small openings were intended to reduce the chance of losses during sampling, and the special design of the holes allows withdrawal of the solvent phase after extraction (Figure 5). The tube volume was set at about 37 mL so that the sampling device was easy to handle. To sample pure alveolar air, the tube volume must be less than the volume of the alveolar air fraction in a single exhaled breath. Since the latter volume is approximately 350 mL [30], this requirement has been met. Due to the small tube volume, the tube is flushed with alveolar air approximately nine times until the sample is accommodated. The manufacturing process of the designed glass tubes is comparable to the manufacturing of disposable, screw-cap, laboratory glass vials. The manufacturing cost should therefore be comparatively low. The choice of the 13–425 screw thread (Glass Packaging Institute/USA) at the tube ends allows the use of common mass-produced screw-vial caps and septa, as well as Mininert valves (Supelco, Bellefonte, USA). The latter could be useful for the preparation of gaseous standards in special cases.

The volumes of the gas sampling tubes varied slightly due to manufacturing. Volume determinations in part of the production batch (*n* = 500) had the following results: mean 37.5 mL, range 37.2–37.8 mL, and RSD 0.29%. All calculations in this paper took account of the tubes' individual volumes. For the purposes of future routine analysis, however, it may often be sufficient to use the mean value and to ignore the volume variations.

#### 3.1.2. End-Exhaled Air Sampling Procedure

Since the blood/air partition coefficient for tetrachloroethene is above a figure of about 10, for example, Koizumi determined a value of 11 at 37°C [31], alveolar air sampling without a breath holding time provides a valid index of the solvent's mixed venous concentration [32]. This assumes, however, that the subject is exhaling after a period of normal ventilation at a constant rate [32]. Unintentionally, however, some subjects spontaneously tend to inhale more deeply to blow into the tube. In these cases, gas equilibration in the lung is slowed [32]. A breath holding time of 5 seconds was therefore chosen for the sampling procedure.

#### 3.1.3. Solvent Extraction

Before development of the solvent extraction method could commence, a suitable solvent had to be selected. The solvent had to meet the following criteria:

- miscibility with the analyte,
- immiscibility with water,
- lowest possible vapour pressure,
- significantly lower retention time than the analyte in gas chromatography,
- no interfering impurities.

Isooctane satisfies these criteria and was therefore chosen as the extraction solvent. It is miscible with tetrachloroethene but immiscible with water. The latter is important because breath samples in the gas sampling tubes always contain condensed water. For example, the results of the field study in Section 3.3 showed a median value of 33 mg of water per tube. The relatively low vapour pressure of isooctane reduces solvent losses during analysis and increases the robustness of the method. Blank isooctane samples showed no interfering signals in chromatographs. Under the selected chromatographic conditions, isooctane has a significantly lower retention time than tetrachloroethene: 3.1 min versus 5.2 min. Isooctane therefore does not interfere with the analyte signal or extend the analysis time.

The isooctane volume used for the extraction process should be as small as possible in order to achieve a high level of analyte enrichment yet also large enough to achieve good extract recovery. Against this background, the solvent volume was set at 200 *μ*L. The use of the roller mixer in the extraction step leads to the formation of a film of isooctane on the glass wall with a large surface area for gas absorption. Partition equilibrium is therefore reached quickly. Isooctane has a lower density (0.69 g/mL) than water, but the isooctane phase collects at the bottom of the gas sampling tubes if the tubes are held up vertically (Figure 4). The condensed water adheres to the glass wall, mainly in a thin dispersion. It is therefore possible to separate the extract using a syringe (Figure 5). About 50 *μ*L of the extract can usually be transferred into the gas chromatography vials. Water very rarely entered the syringe; when it did, two phases were visible in the vials. The water phase was then simply removed using a syringe.

#### 3.1.4. Gas Chromatographic Analysis

Gas chromatographic analysis of the solvent extract of an end-exhaled air sample is very simple and is completed in about 7 minutes. Excellent chromatograms were obtained using a standard column (HP-1, 30 m), as shown in Section 3.3. Since only a very small quantity of the solvent extract is used for analysis (only 2 *μ*L in the proposed method), a sample extract can be analysed repeatedly. Moreover, the extracts can be diluted if the analyte concentration value exceeds the upper limit of the working range. In this regard, therefore, the use of liquid extracts has advantages over direct exhaled air analysis.

#### 3.1.5. Working Range of the Method

The working range of the analytical method must cover the suggested limit value of 3 ppm tetrachloroethene in end-exhaled air [3, 4]. Since today's typical concentrations in workers at dry-cleaning shops are well below that limit value, the method should also cover these levels; for example, McKernan et al. measured a preshift value of 0.51 ppm (arithmetic mean, 18 subjects from four shops) [18]. The working range was therefore defined as 2 to 20 *μ*g/L, corresponding to about 0.3 to 3 ppm.

### 3.2. Method Performance Evaluation

#### 3.2.1. Calibration

**Figure 6:**
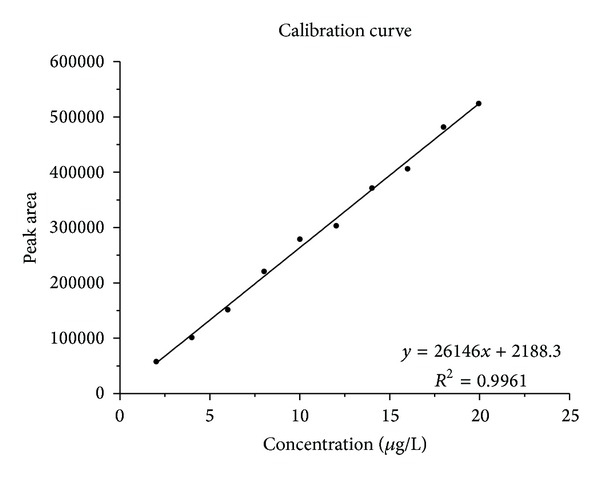
Calibration curve for the determination of tetrachloroethene in end-exhaled air; peak area against concentration used.

**Figure 7:**
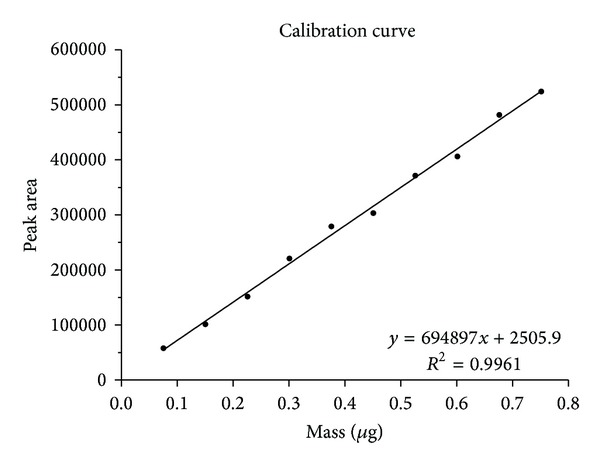
Calibration curve for the determination of tetrachloroethene in end-exhaled air; peak area against mass used.

Calibration curves were obtained by plotting the peak areas of tetrachloroethene against the concentrations or masses used. Representative calibration curves are shown in Figures 6 and 7.

The curves are linear within the investigated working range of the method (between 2 and 20 *μ*g tetrachloroethene per litre of exhaled air). High values were obtained for the coefficient of determination (*R* ^2^ > 0.99). In the calibration curve procedure for determining the limit of detection in Section 2.2.5, one calibration was shifted to a lower concentration range (0.005 to 0.05 *μ*g/L). The curve thus obtained was also linear, with a coefficient of determination of 0.99. The method of calibration is therefore acceptable and can easily be adapted to other concentration ranges.

#### 3.2.2. Method Precision

The intraday precision was determined at the concentration levels 4 and 15 *μ*g tetrachloroethene per litre of end-exhaled air. The results are presented in Table 2 and show that the precision expressed as relative standard deviation is less than 7%. It must be noted that the stated precision includes variations arising through sample preparation, especially with regard to the tetrachloroethene spiking procedure.

#### 3.2.3. Accuracy

Reference materials were not available and there was no possibility of interlaboratory comparability investigations. Spiked end-exhaled air samples were therefore used for recovery experiments at two concentration levels. The results are presented in Table 2. Slightly less tetrachloroethene was recovered than the calculated additions, but the recovery rates are reproducible and sufficient for routine biomonitoring measurements. In addition, the spiking gas was prepared using a static method. Adsorption effects [33] in the static dilution bottle and the syringes may decrease the actual tetrachloroethene concentration in the spiking gas and subsequently lead to an overestimation of the method's inaccuracy.

The use of an internal standard, for example, ^13^C tetrachloroethene, might improve the reliability of the method, including the recovery. The extra effort for the internal standard procedure could be considered particularly in nonroutine applications which are beyond the aim of this work.

#### 3.2.4. Limits of Detection and Quantification

The limits of detection and quantification of tetrachloroethene in end-exhaled air were determined using the calibration curve procedure and spiked end-exhaled breath samples. A realistic extraction step was therefore included in the experiment.

Under the given conditions for sample preparation and gas chromatographic determination, the limit of detection was 0.005 *μ*g/L and the limit of quantification was 0.02 *μ*g/L. The limit of quantification is therefore one-thousandth of the suggested biological assessment values [3, 4] and shows the capability of the developed method.

#### 3.2.5. Stability of End-Exhaled Air Samples

**Figure 8:**
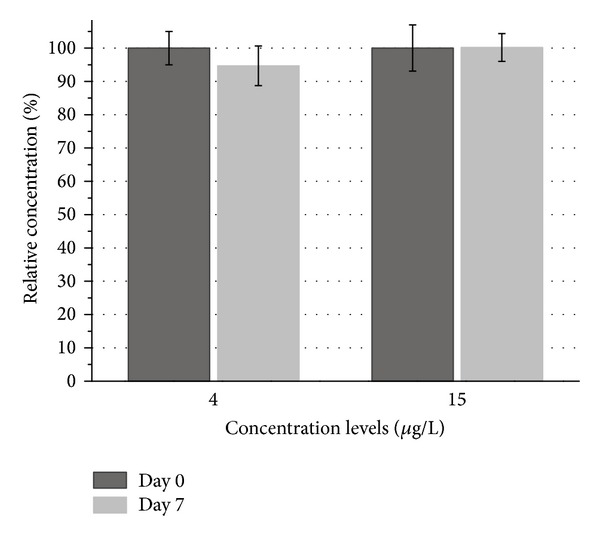
Results of the storage test of end-exhaled air samples over seven days; *n* = 10 per level per day; mean concentration of the start day (Day 0) defined as 100%.

Exhaled air from workers exposed to tetrachloroethene is commonly sampled on the last day of a working week [3]. The samples are then sent to the laboratory by standard post. Typically, the laboratory receives the samples after the weekend, so they must remain stable for at least three days at ambient temperature. The stability of breath samples was therefore determined for a storage time of one week at room temperature.  Figure 8 shows the results of the stability test. Whereas no tetrachloroethene loss was observed for the concentration level 15 *μ*g/L, a very slight loss of 5% was obtained for the level 4 *μ*g/L. The samples therefore remain sufficiently stable for one week. This conclusion is supported by other authors, who have reported breath sample stability for at least five days in glass tubes [5, 22].

### 3.3. Field Study

A field study was conducted to verify the applicability of the proposed method.  Table 3 shows the results of the end-exhaled air analyses of subjects exposed to tetrachloroethene (*n* = 4) and nonexposed subjects (*n* = 10, control group).

The sampling procedure was easy to apply and was accepted well by all subjects. Double sampling, in which the subjects filled two gas sampling tubes consecutively, took about 4 minutes. The water content in the tubes ranged from 14 to 60 mg (median 33 mg, *n* = 28).

**Figure 9:**
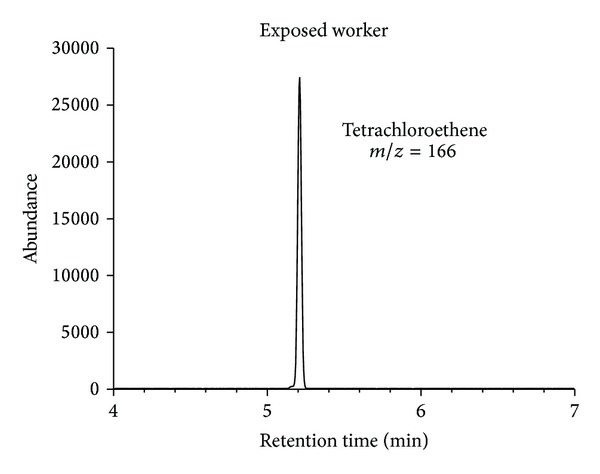
Representative chromatogram of an end-exhaled air sample from a dry-cleaning worker (machine operator) exposed to tetrachloroethene; tetrachloroethene peak (retention time 5.2 min,* m/z* 166).

**Figure 10:**
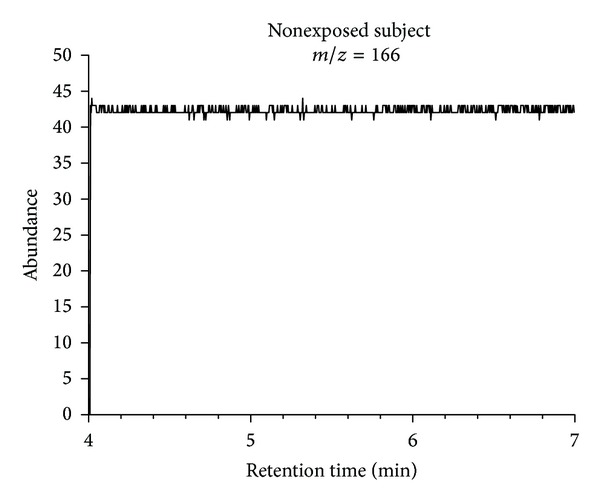
Representative chromatogram of an end-exhaled air sample from a nonexposed subject (control group).

Representative chromatograms of solvent extracts from end-exhaled breath samples from the study participants are shown in Figures 9 and 10.

None of the chromatograms showed any interfering matrix signals. The method therefore exhibits excellent selectivity. The chromatograms obtained for samples from the dry-cleaning workers showed well-shaped tetrachloroethene peaks. In contrast, no significant peaks were observed in the chromatograms for the nonexposed subjects.  Table 3 shows excellent agreement between the results for both samples (A and B) from the participants. The level of tetrachloroethene ranged from 3.4 to 16.7 *μ*g/L for the exposed workers. Here, the ion ratios* m/z* 166 to* m/z* 131 (target to qualifier ion) for the tetrachloroethene peak agreed between the standards and samples.

When the exhaled air leaves the mouth of a subject during the sampling procedure, the exhaled air will adapt to the ambient pressure and to the temperature of the glass tube, which is commonly equivalent to the ambient temperature. It can be assumed that this adaptation is complete by the time the tube is closed. Measuring the ambient pressure and temperature therefore allows the measured concentrations to be converted to standard conditions (1013 hPa/20°C). This conversion decreases the concentrations stated in Table 3 by 3%. The need to convert the concentrations arises from the respective accuracy requirements. For routine analysis, it may often be acceptable to ignore the real pressure and temperature conditions.

The tested method therefore allows determination of occupational exposure levels and distinction between occupationally exposed and nonexposed subjects.

## 4. Conclusions

The desirable routine use of noninvasive exhaled air analysis in occupational biological monitoring requires simple sampling procedures that are suitable for field use and analysis methods that can be performed in common biomonitoring laboratories. The method developed here fulfills these requirements: end-exhaled air sampling is performed using the classical glass tube technique. A special tube design, developed and used within this study, enables reproducible sampling and means that the tubes can be used directly as separating funnels. The latter allows a simple transformation of end-exhaled air samples into “liquid samples” using a microscale solvent extraction. The liquid samples can be analysed using a common gas chromatography system. A simple liquid autosampler allows the analytical step to be automated. Since the calibration procedure is based on liquid standards, there is no need to prepare gaseous standards.

The method's concept was successfully applied to the determination of tetrachloroethene in end-exhaled air, which acts as a biomarker for occupational tetrachloroethene exposure. Validation experiments demonstrated acceptable sensitivity, selectivity, precision, and accuracy in the analytical method. A field study proved the applicability of the method, which addresses typical end-exhaled air concentrations from exposed workers. The method can be deployed rapidly, requires no previous experience in gas analysis, and seems to be easily transferable to other workplace chemicals.

## Figures and Tables

**Table 1:** Example pipetting scheme for the preparation of the calibration samples; concentration range 2 to 20 *μ*g tetrachloroethene/L exhaled air.

Number	Calibration solutions				Calibration samples			
	Conc. stock solution II (mg/L)	*V* stock solution II (*μ*L)	*V* final (mL)	Conc. (µg/L)	*V* calibration solution (µL)	*V* gas sampling tube (mL)	Mass (µg)	Conc. (µg/L)
1	93.9	40	10	376	200	37.433	0.0751	2.0
2	93.9	80	10	751	200	37.668	0.1502	4.0
3	93.9	120	10	1127	200	37.639	0.2254	6.0
4	93.9	160	10	1502	200	37.524	0.3005	8.0
5	93.9	200	10	1878	200	37.573	0.3756	10.0
6	93.9	240	10	2254	200	37.486	0.4507	12.0
7	93.9	280	10	2629	200	37.544	0.5258	14.0
8	93.9	320	10	3005	200	37.593	0.6010	16.0
9	93.9	360	10	3380	200	37.598	0.6761	18.0
10	93.9	400	10	3756	200	37.661	0.7512	19.9

**Table 2:** Method precision and accuracy expressed as relative standard deviation and mean recovery, respectively; determined using 10 individual samples in each case.

Concentration levels (µg/L)	Precision RSD (*n* = 10) (%)	Mean recoveries (*n* = 10) (%)
4	4.8	83.9
15	6.9	82.7

**Table 3:** Measurement results of the field study: tetrachloroethene in end-exhaled air in exposed (dry-cleaning workers) and nonexposed (control group) subjects.

Subject			Tetrachloroethene conc. in end-exhaled air		Sampling conditions	
		Activity	Sample A	Sample B	*p*	Temp.
Number	Status		(µg/L)		(hPa)	(°C)
1	Exposed	Dyeing	7.6	7.9	1010	9.4
2	Exposed	Machine operating	16.7	16.7	1010	9.4
3	Exposed	Tagging/inspecting	5.9	5.6	1010	9.4
4	Exposed	Pressing	3.4	3.5	1011	11.0
5–14	Control	—	<LOQ	<LOQ	1016 to 1020	11.5 to 18.0

*p*: ambient pressure; Temp.: ambient temperature.
